# Host Species Affects Gut Microbial Community and Offspring Developmental Performances in the Pupal Parasitoid *Chouioia cunea* Yang (Hymenoptera: Eulophidae)

**DOI:** 10.3390/insects15090722

**Published:** 2024-09-20

**Authors:** Lina Pan, Jiamin Liao, Yiping Hu, Rui Ren, Wei Chen, Zixin Liang, Fan Lu, Meidi Sun, Zhiqin Song, Xiaoyu Li, Weiyi Zhang, Wenfang Gao, Chuncai Yan, Min Li

**Affiliations:** Tianjin Key Laboratory of Conservation and Utilization of Animal Diversity, Tianjin Normal University, Tianjin 300387, China; skypln@tjnu.edu.cn (L.P.); 13974602523@163.com (J.L.); hypdyouxiang@163.com (Y.H.); renrui15222212976@163.com (R.R.); 15620272533@163.com (W.C.); 13362516302@163.com (Z.L.); lufan06071809@163.com (F.L.); 15122532198@163.com (M.S.); 17658382256@163.com (Z.S.); lxiaoyu0406@163.com (X.L.); 15222009030@163.com (W.Z.); skygwf@tjnu.edu.cn (W.G.)

**Keywords:** *Chouioia cunea* Yang, host, parasitism, intestinal microorganism

## Abstract

**Simple Summary:**

*Chouioia cunea* Yang is a crucial natural enemy in the management of the fall webworm (*Hyphantria cunea*), which is a globally significant quarantine pest. The feeding behavior, dispersal, and high reproductive capacity of *Hy. cunea* cause substantial economic and ecological damage. Apart from *Hy. cunea*, *C. cunea* also exhibits parasitic behavior toward various Lepidoptera species, including those from the families Lasiocampidae, Lymantridae, Notodontidae, Psychidae, and Noctuidae. It also targets certain Coleoptera species, such as those from the families Chrysomelidae and Coccinellidae, most of which are leaf-eating pests. *C. cunea* feeds on the hemolymph of its host. Variations in the hemolymph’s nutrient content, chemical properties, and immune response among different host species may influence the gut microbiome of the wasps. Moreover, microbial communities present in the host can be ingested by the parasitic wasps, leading to differences in their gut microbiome compositions depending on the host. In this study, we investigated the developmental patterns and intestinal microbiota diversity of *C. cunea* parasitizing various hosts. Our objective was to elucidate how different hosts impact the development and intestinal microbiota composition of *C. cunea*. This research provides scientific evidence for optimizing biological control strategies and applications involving insect pathogenic microorganisms.

**Abstract:**

*Chouioia cunea* are known to exploit in varying degrees a wide range of lepidopteran species and its offspring development may vary with host species. This study examined its preimaginal development and larval gut microbiota in parasitizing five folivorous lepidopteran hosts including *Hyphantria cunea* (referred to thereafter as CcHc), *Antherea pernyi* (CcAp), *Helicoverpa armigera* (CcHa), *Spodoptera exigua* (CcSe), and *Spodoptera frugiperda* (CcSf). Though rates of parasitism and offspring eclosion did not change with host species, the development period and number of offspring eclosed varied with hosts, with the shortest period in CcSf and the highest number from CcAp. For offspring larval gut microbiota, though phylum Proteobacteria was dominant for attacking CcAp, Firmicutes was so for the other hosts. All microbial genera except *Enterococcus* were less abundant for CcSf than the other hosts. The database-based predictions indicate a significant positive correlation between *Cutibacterium* and *Aureimonas* with the relative number of wasp emergence, while *Blastomonas* exhibits a strong positive association with the developmental period. Our results imply the potential relevance of the gut microbial community in offspring larvae to host species attacked by *C. cunea*.

## 1. Introduction

Insects represent the most diverse and abundant group of animals, and their diversity and evolution are, to some extent, influenced by their interactions with microorganisms [1]. Microorganisms, particularly gut microbiota, play crucial roles in host development, reproduction, behavior, and stress resistance [1,2,3]. Bacterial communities are essential for the maintenance of host health and the immune system, while dietary sources significantly impact the diversity of the insect gut microbiota [4,5,6]. Extensive research has been conducted on herbivorous insects such as the fall armyworm *S. frugiperda* [7,8], the peach fruit moth *Carposina sasakii*, the oriental fruit moth *Grapholita molesta* [9], and the diamondback moth *Plutella xylostella* [10], as well as predatory insects such as orb-weaver spiders *Eriovixia cavaleriei* and *Larinioides cornutus*, the hunting spider *Pardosa pseudoannulata* [11] and predaceous ladybirds *Harmonia axyridis* [12]. However, our understanding of parasitic insects remains limited. 

Limited research on the intestinal microbiota in parasitic insects can be seen in the parasitic wasps *Asobara japonica* (Hymenoptera; Braconidae: Alysiinae) [13] and *Nasonia vitripennis* (Hymenoptera; Pteromalidae) [14]. The microbiome composition of the solitary parasitic wasp *A. japonica*, which can parasitize various Drosophila species, is influenced by geography and population structure but not by the presence of the endosymbiont *Wolbachia* [13]. *N. vitripennis* targets the pupae of species from the families Calliphoridae, Muscidae, and Sarcophagidae [14]. Different hosts, such as *Lucilia sericata*, *Sarcophaga marshalli*, and *Musca domestica*, significantly impact the bacterial communities of their corresponding parasitoid *N. vitripennis*, resulting in variations in the microbial community’s diversity [14]. Based on these studies, it can be inferred that the microbiome composition of parasitoid wasps, such as *A. japonica* and *N. vitripennis*, is significantly influenced by geographical factors, population structure, and the specific host species they parasitize. However, there has been limited research on the association of these disparities with their growth and development.

The parasitoid wasp *Chouioia cunea* Yang is a primary natural enemy used for the biological control of the fall webworm *Hyphantria cunea*. Additionally, it serves as an endo-parasitoid of the pupal stage of various lepidopteran insects, encompassing those from diverse families such as Lasiocampidae, Lymantridae, Notodontidae, Psychidae, Noctuidae, Bombycidae, Saturniidae, Yponomeutidae, Hepialidae, Pyralididae, Geometridae, and Pieridae. Furthermore, it targets certain Coleoptera species including those from Chrysomelidae and the Coccinellidae families [15,16]. Our previous study found that *C. cunea* can parasitize the pupae of its natural hosts (*Hyphantria cunea* and *Helicoverpa armigera*) and non-natural hosts (*Spodoptera exigua* and *Spodoptera frugiperda*); however, the number of emerging adults was significantly higher in natural hosts than in non-natural hosts [17]. As an endoparasitoid, the eggs, larvae, and pupae of *C. cunea* inhabit the host pupa throughout their life cycle, emerging as adults by biting through the host pupal shell. This enclosed environment provides ample opportunities for *C. cunea* to interact with the host’s microbial community. This observation led us to postulate that the host’s nutritional status and microbial composition substantially influence the development of *C. cunea*. However, there is little research on the effects of varying host pupae on the microbial community structure and diversity of *C. cunea*. 

In our study, we employed high-throughput sequencing of the V3–V4 hypervariable region of the 16S rDNA to investigate the impacts of different hosts *Hyphantria cunea* (the natural host), *Antherea pernyi* (the substitute host), *Helicoverpa armigera* (another natural host), *Spodoptera exigua* (a non-host), and *Spodoptera frugiperda* (another non-host) on the gastrointestinal microbiota of *C. cunea*. This study provides insights into how different hosts influence the gut microbiota of parasitic wasps and potentially affect their growth and development. This provides a foundation for more effectively using parasitic wasps to control various agricultural and forestry pests.

## 2. Materials and Methods

### 2.1. Insect Rearing and Development Study

The parasitoid wasp *Chouioia cunea* was obtained from the Natural Enemy Breeding Center of Luohe Central South Forestry (Luohe, China). The tussah silkworm, *Antheraea pernyi*, served as the substitute host for *C. cunea* and was sourced from the Benxi Tussah Breeding Base in Benxi, China. The pupae of *A. pernyi* were placed in conical flasks, each sealed with nylon mesh, at a ratio of one pupa to forty *C. cunea* adults. The cultures were maintained in an incubator at 25 °C with 70% relative humidity (RH) in total darkness. After 3 days, the conditions were adjusted to 25 °C with 75% RH under a 14:10 light–dark cycle. The cultures were incubated until adult emergence, occurring within 17 to 20 days.

In the developmental experiment, pupae of five lepidopteran species, namely *Hyphantria cunea* (Lepidoptera: Erebidae), *Antherea pernyi* (Lepidoptera: Saturniidae), *Helicoverpa armigera* (Lepidoptera: Noctuidae), *Spodoptera exigua* (Lepidoptera Noctuidae), and *Spodoptera frugiperda* (Lepidoptera: Noctuidae) were exposed to parasitism by *C. cunea* offspring from the same pupa of *A. pernyi*. The CcHc group, utilizing *Hy. cunea* pupae as hosts, was replicated three times with 20 pupae inoculated in each replicate. The CcAp group, using *A. pernyi* pupae as hosts, was also replicated three times with 10 pupae inoculated in each replicate. Similarly, the CcHa group (*He. armigera*), the CcSe group (*S. exigua*), and the CcSf group (*S. frugiperda*) were all replicated three times with 14 pupae inoculated in each replicate for their respective host species. The cultures were initially maintained in an incubator at 25 °C with 70% relative humidity (RH) in complete darkness. After 3 days, they were transferred to an incubator at 25 °C with 70% relative humidity and a 14:10 h (L–D) photoperiod until the wasps emerged naturally. We measured the following developmental parameters: parasitism rate as the proportion of offspring parasitoids eclosed from a host in total hosts exposed to parasitism, and emergence rate as the proportion of hosts producing adult parasitoids in those being parasitized. To control for the effect of host body mass, we employed the body weight of *Hy. cunea* as the basic unit (denominator) to divide the body mass of the other hosts to obtain respective ratios, which were then used to multiply the number of offspring to obtain a measure relative to body mass. Correlation analyses were calculated using one-way ANOVA and the SPSS software, version 26.0 (IBM Corp., released 2019; IBM SPSS Statistics for Windows, Version 26.0. IBM Corp.: Armonk, NY, USA).

### 2.2. Sample Collection and DNA Extraction

The wasps were split into five batches. Each batch was reared on *Hy. cunea* (named CcHc), *A. pernyi* (named CcAp), *He. armigera* (named CcHa), *S. exigua* (named CcSe), and *S. frugiperda* (named CcSf) pupae. The larvae of *C. cunea* were extracted from the host pupa after parasitizing for 10–12 days. The insect surface was wiped with 70% alcohol for 30 s, soaked in 0.25% sodium hypochlorite for 1 min, and then rinsed three times with sterile water to remove external contaminants. In a sterile environment, the insect abdomen was opened with sterilized forceps, and the entire intestine was extracted and immediately rinsed twice with 0.9% sterile NaCl solution. The intestines were then placed in a centrifuge tube containing 600 μL of sterile PBS, and 200–300 intestines were mixed as a sample. There were five groups—CcHc, CcAp, CcHa, CcSe, and CcSf—with three samples in each group. After quick centrifugation and supernatant removal, the samples were rapidly frozen in liquid nitrogen and sent to Shanghai Majorbio Bio-pharm Technology Co., Ltd. (Shanghai, China) for sequencing.

Total microbial genomic DNA was extracted using the DNeasy^®^ PowerSoil^®^ Pro Kit (Qiagen, Hilden, Germany) according to the manufacturer’s instructions. The DNA purity and concentration were determined using a NanoDrop 2000 UV–vis spectrophotometer (Thermo Scientific, Wilmington, NC, USA), followed by 1% agarose gel electrophoresis to assess the DNA integrity.

### 2.3. 16S rRNA Gene Amplification and Sequencing

The V3–V4 hypervariable regions of the bacterial 16S ribosomal RNA (rRNA) gene were amplified with the primer pairs 338 F (5′-ACTCCTACGGGAGGCAGCAG-3′) and 806 R (5′-GGACTACHVGGGTWTCTAAT-3′) to analyze the gut microbiota of *C. cunea* fed on different hosts. The PCR reaction mixture consisted of 4 µL of 5× FastPfu reaction buffer, 2 µL of 2.5 mM dNTPs, 0.4 µL of TransStart FastPfu Polymerase, 0.8 µL of 5 µM of each primer, 0.2 µL of BSA, 10 ng of the template DNA, and DNA-free water to a final volume of 20 µL.

The PCR reaction included a single denaturation step at 95 °C for 3 min, followed by 30 cycles at 95 °C for 30 s, 55 °C for 30 s, 72 °C for 45 s, and a single extension at 72 °C for 10 min. The PCR products were separated on 2% (*w*/*v*) agarose gel. Those with the correct size were excised and purified using an AxyPrep DNA gel extraction kit (Axygen Biosciences, Union City, CA, USA). 

For the purified 16S PCR products, Illumina MiSeq sequencing libraries were created using the TruSeqTM DNA Sample Prep Kit (San Diego, CA, USA) and then sequenced on Illumina MiSeq (San Diego, CA, USA) according to the standard protocols.

### 2.4. OTU Identification and Diversity Analysis

The raw data from the Illumina MiSeq sequencing were demultiplexed using an in-house perl script to obtain the sequencing data for each sample. The quality of the raw data was checked by using Fastp version 0.19.6 [18]. Paired-end reads were merged using Flash version 1.2.11 with the following criteria: (i) The reads were truncated at any site receiving an average quality score of <20 over a 50 bp sliding window, truncated reads shorter than 50 bp were discarded, and reads containing ambiguous characters were also discarded. (ii) Only overlapping sequences longer than 10 bp were assembled according to their overlapped sequence. The maximum mismatch ratio of the overlap region was 0.2. Reads that could not be assembled were discarded. (iii) Samples were distinguished according to the barcode and primers, and the sequence direction was adjusted with the exact barcode matching and two nucleotide mismatches in the primer matching. Operational taxonomic units (OTUs) were clustered with a 97% similarity threshold using Uparse version 11. Chimeric sequences were identified and removed using the Uchime algorithm in Usearch version 11. The taxonomy of each 16S rRNA gene sequence was analyzed with a naïve Bayesian classifier of the Ribosomal Database Project version 2.13 against the silva138/16s_bacteria database. 

### 2.5. Statistical Analysis

Bioinformatic analysis was carried out using the Majorbio Cloud platform (https://cloud.majorbio.com, accessed on 16 February 2023). Based on the OTUs information, rarefaction curves, and alpha diversity indices, including the observed OTUs, Chao1 richness, Shannon index, and Good’s coverage, were calculated with Mothur (version v.1.30.2 https://mothur.org/wiki/calculators/, accessed on 2 October 2009) [19]. The similarity between the microbial communities in different samples was determined using principal component analysis (PCA) and non-metric multidimensional scaling (NMDS) based on Bray–Curtis dissimilarity using the Vegan v2.5-3 package [20]. The PERMANOVA test was used to assess the percentage of variation explained by the treatment along with its statistical significance using the Vegan v2.5-3 package [20]. 

## 3. Results

### 3.1. The Parasitic Capability and Development of C. cunea among Different Hosts

**Figure 1:**
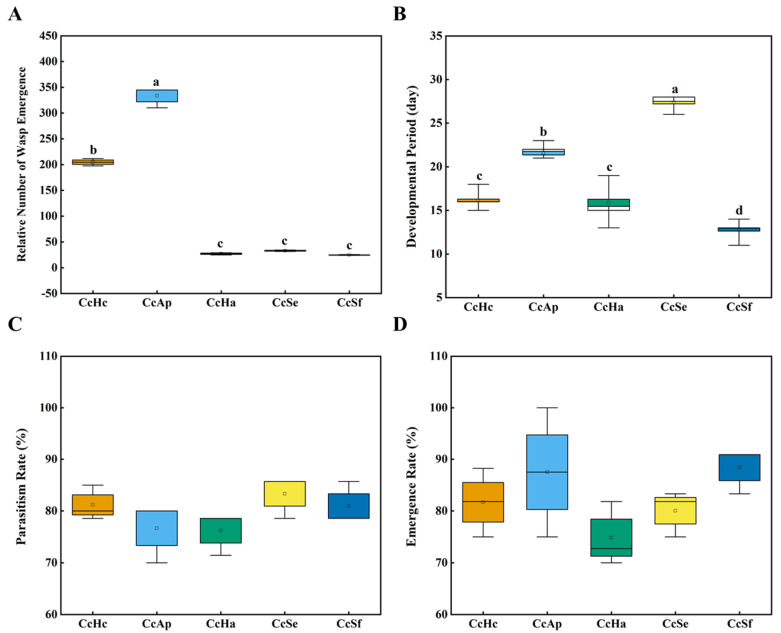
The number of parasitoid offspring that emerged from a single host (**A**), egg−to−adulthood developmental period (**B**), parasitism rate (**C**), and offspring emergence rate (**D**) regarding different hosts in *C. cunea*. CcHc stands for *Hy. cunea*, CcAp stands for *A. pernyi*, CcHa stands for *He. armigera*, CcSe stands for *S. exigua*, and CcSf stands for *S. frugiperda*. The sample size of CcHa, CcSe, and CcSf was 14, and the sample size of CcHc 20 and CcAp was 10. The different letters above the box indicate a statistical difference between hosts (*p* < 0.05 on two tails).

We divided the wasps into five groups and reared them on pupae from *Hy. cunea* (CcHc), *He. armigera* (CcHa), *A. pernyi* (CcAp), *S. exigua* (CcSe), and *S. frugiperda* (CcSf) to investigate the effects of different hosts on the development of *C. cunea*. As shown in Figure 1A, the relative number of wasp emergences in CcAp was the highest, followed by CcHc; however, there were no significant differences among the remaining three groups. Regarding the development periods, the time from parasitism to adult emergence for wasps in CcSf was approximately 13 days. Conversely, CcSe exhibited the longest developmental period, taking up to 28 days, followed by CcAp, which required 22 days. Additionally, both CcHc and CcHa had a developmental period of approximately 16–18 days (Figure 1B). However, the parasitism rates and emergence rates of *C. cunea* did not significantly differ across these samples (Figure 1C,D). In summary, different hosts did not result in significant variations in the parasitism rate and emergence rate of *C. cunea*; however, the number of emerging wasps and the duration of development significantly differed.

### 3.2. Sequencing Data of 16S rRNA

As reported, the gut microbiota of parasitic insects is influenced by their hosts, and different hosts may lead to changes in the gut microbiota of the parasitic organism, thereby impacting its ecological adaptability [14]. We analyzed the gut microbiota of *C. cunea* parasitizing five different host pupae using high-throughput sequencing of the V3–V4 hypervariable region of the 16S rDNA to investigate the influence of different host pupae on the gut microbiota of *C. cunea*. A total of 644,231 optimized sequences and 274,682,443 bases were obtained, with an average sequence length of 426 bp. These sequences were clustered into 1229 OTUs at a 97% sequence identity using the USEARCH11-uparse algorithm. The sequencing data statistics for all samples are provided in Table S1. At the phylum level, the top five phyla were Firmicutes, Proteobacteria, Actinobacteriota, Bacteroidota, and Patescibacteria. At the genus level, the top five genera included were *Enterococcus*, *Achromobacter*, *Lactobacillus*, *Staphylococcus*, and *Pedobacter*. The rarefaction curves for all samples flattened, indicating that the sequencing effectively covered the actual bacterial diversity (Figure S1).

### 3.3. Gut Microbial Diversity of C. cunea among Different Hosts

**Figure 2:**
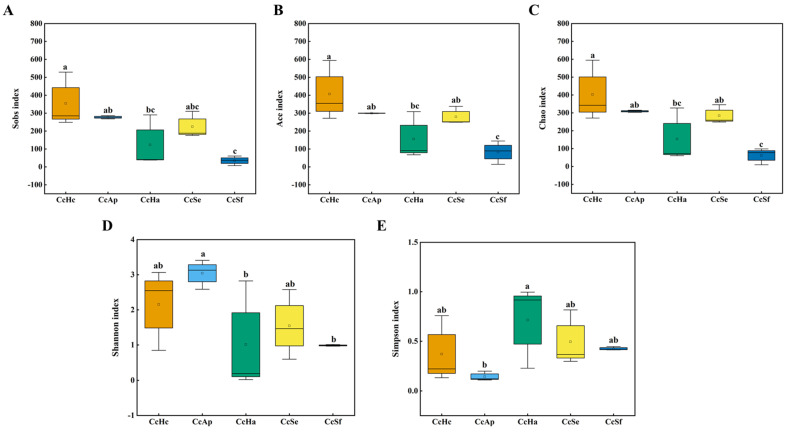
Alpha diversity of gut microbiota in *C. cunea* among different hosts. Sob index (**A**), Ace index (**B**), Chao index (**C**), Shannon index (**D**), and Simpson index (**E**) on the OTU level of gut bacterial microbiota among CcHc, CcAp, CcHa, CcSe, and CcSf. The abscissa represents the sample names, and the ordinate represents the values of a certain index type observed at the selected taxonomic level. The different letters above the box indicate a statistical difference, as determined using one-way ANOVA (*p* < 0.05). Groups labeled with the same letter indicate no significant difference, while groups labeled with different letters denote a statistically significant difference.

Regarding alpha diversity, the Ace indices did not significantly differ, which reflects microbial community richness among CcHc, CcAp, and CcSe. However, a significant difference was observed in the Sobs, Ace, and Chao indices between CcSf and both CcHc and CcAp (Figure 2A–C). The Simpson and Shannon diversity indices exhibited a significant disparity between CcAp and CcHa, indicating that the CcAp sample harbored a more diverse microbial community than the CcHa sample. Conversely, no statistically significant variations were observed in the Shannon indices between CcHc, CcHa, and CcSe (Figure 2D,E). 

**Figure 3:**
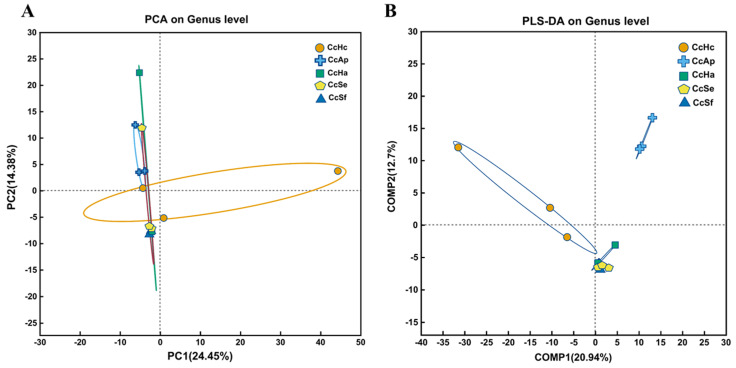
Beta diversity and sample group analysis of gut microbiota in *C. cunea* among different hosts. PCA (principal component analysis) (**A**) and PLS-DA (partial least squares discriminant analysis) (**B**) at the genus level of gut bacterial microbiota among CcHc, CcAp, CcHa, CcSe, and CcSf.

The PCA analysis is depicted in Figure 3A, where PC1 accounts for 24.45% of the original dataset’s features. PC1 showed no significant separation in the gut microbiota of *C. cunea* larvae parasitizing different hosts. However, a discernible separation between CcAp and CcSf was observed in the second principal component (PC2), which explained 14.38% of the original dataset’s features. We employed PLS-DA (partial least squares discrimination analysis) to analyze sample variations to maximize intergroup dissimilarities. As depicted in Figure 3B, COMP1 represented 20.94% of the original dataset’s features. Based on COMP1, CcHc exhibited a distinct separation from other samples within our analysis set. The inclusion of COMP2 in the analysis revealed an additional 12.7% of the original dataset’s features and highlighted that both CcHc and CcAp exhibited distinct separation from the other groups within this specific principal component. This finding is consistent with the observed variations in relative wasp emergence rates.

### 3.4. Gut Microbial Composition of C. cunea among Different Hosts

**Figure 4:**
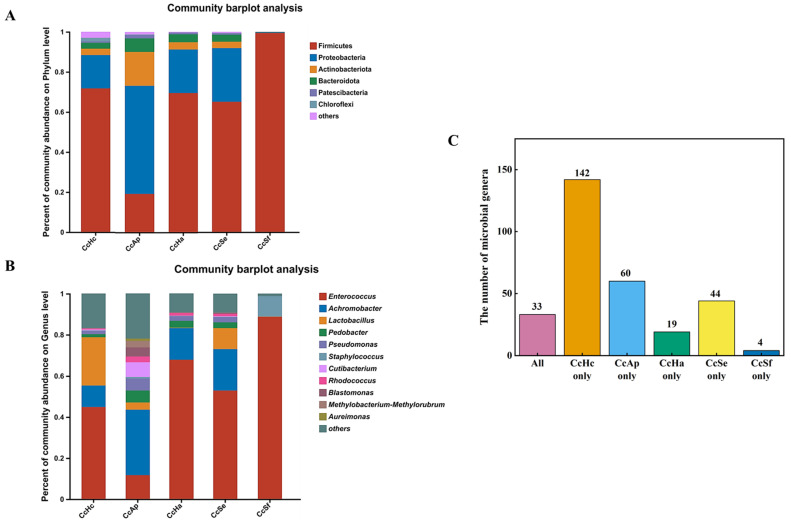
Bacterial composition analysis. Column plots of communities of different samples at phylum (**A**) and genus (**B**) levels. The *x*-axis represents the sample names, while the *y*-axis represents the proportion of species in the sample. The different colors of the bars represent different species, and the length of each bar represents the proportion of each species. The unique and common genera of bacteria in each sample of the Venn diagram at the genus level (**C**).

At the phylum level, Firmicutes was the dominant group of gut bacteria in CcSf, CcHa, CcHc, and CcSe, accounting for 99.4%, 69.5%, 71.8%, and 65.1%, respectively (Figure 4A). The CcHa, CcHc, and CcSe samples also had Proteobacteria as the second dominant phylum of gut microbes. In the case of CcAp, the predominant bacterial phylum in the gut was Proteobacteria, accounting for 54%, while Firmicutes (19.2%) and Actinobacteriota (16.9%) were the second and third most prevalent phyla, respectively (Figure 4A).

At the genus level, the dominant genus of gut bacteria in CcSf, CcHa, CcHc, and CcSe was *Enterococcus*, while it was *Achromobacter* (31.8%) in CcAp. CcSf had a relatively simple gut microbial composition, with the dominant genus *Enterococcus* constituting 88.7% and the second genus, *Staphylococcus*, accounting for 9.9%, together representing 98.6% of all microbes (Figure 4B). In contrast, CcAp had the most complex gut microbiota, with the dominant genus *Achromobacter* and other genera, such as *Enterococcus*, *Cutibacterium*, *Pseudomonas*, *Pedobacter*, and *Lactobacillus*, accounting for 11.7%, 7.2%, 5.8%, 5.8%, and 3.5%, respectively. CcHc and CcAp, the non-core dominant bacterial groups in the gut, represented the majority, comprising 16.9% and 21.9%, respectively. In contrast, CcHa and CcSe accounted for 9.2% each. CcSf, a non-core species, constituted only 1.2% (Figure 4B). In summary, the most abundant genus found in CcSf, CcHa, CcHc, and CcSe was *Enterococcus*. *Achromobacter* was the most abundant genus for CcAp. The co-occurrence relationships of the core bacteria analysis among CcHc, CcAp, CcHa, CcSe, and CcSf indicated the presence of *Enterococcus* (the most abundant genus) across all samples. *Achromobacter* (the second most abundant genus), *Pedobacter* (the fourth most abundant genus), and *Pseudomonas* (the fifth most abundant genus) were present in all samples except for CcSf. *Lactobacillus* (the third most abundant genus) was found in CcHc, CcAp, and CcSe. (Figure S2).

Taxonomically, the species annotation results identified 44 phyla and 98 classes. These classes were further subdivided into an extensive array of orders (*n* = 218), families (*n* = 355), genera (*n* = 628), and species (*n* = 933). The gut microbiota of CcHc exhibited the highest species diversity, while that of CcSf had the lowest. At the phylum level, 43 phyla (97.7%) were found in CcHc samples, while 22, 20, and 21 phyla were found in CcAp, CcHa, and CcSe, respectively. Only 12 phyla were found in CcSf. At the genus level, CcHc and CcAp also had the most numerous, with 431 (68.6%) and 349 (55.5%), respectively. In terms of operational taxonomic units (OTUs), CcHc demonstrated the highest OTU count, comprising 781 OTUs (63.5%), followed by CcAp with 546 OTUs (44.4%), CcSe with 450 OTUs (36.6%), and CcHa with 322 OTUs (26.2%). Notably, CcSf exhibited the lowest count, consisting of only 97 OTUs (7.9%) (Figure S3). The summary of OTU abundance and classification information can be found in Supplementary Table S2.

The Venn diagram illustrates the presence of 33 genera of microorganisms in the five samples, including *Enterococcus*, *Achromobacter*, *Lactobacillus*, *Staphylococcus*, *Rhodococcus*, *Pedobacter*, and *Pseudomonas* as part of the top 10 most abundant genera. CcHc and CcAp demonstrated a diverse gut microbiota with 142 and 60 genera, respectively. In contrast, CcHa and CcSf exhibited relatively fewer species, at 19 and 4 genera, respectively (Figure 4C). The comprehensive data and the original Venn diagram can be found in Supplementary Table S3 and Figure S4.

**Figure 5:**
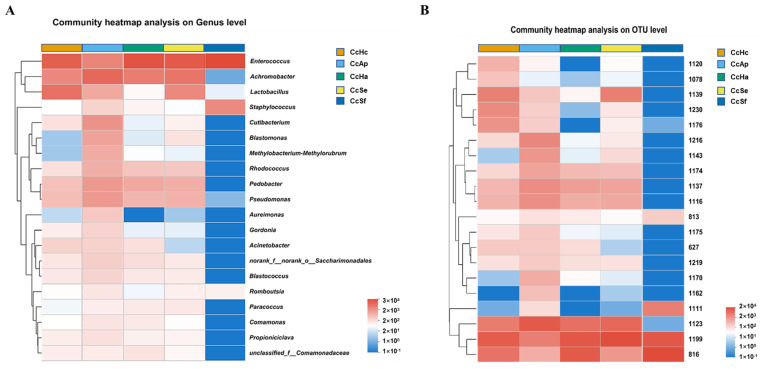
The cluster heat map of the 20 most abundant genera (**A**) and OTU (**B**) in the bacterial community among CcHc, CcAp, CcHa, CcSe and CcSf. The horizontal axis represents the sample names, while the vertical axis represents the species/OTU names. The abundance variation of different species in the sample is shown via the color gradient of the blocks, with the numerical values represented by the color gradient on the right side of the graph.

The heatmap illustrates the relative abundance of the top 20 genera among the five samples. At the genus level, the clustering of gut samples revealed that the gut microbiota of CcSf was distinctly different from the other groups. Among the top five genera, *Enterococcus* was abundant in all five samples, while *Achromobacter* was less prevalent in the gut microbiota of CcSf but more so in the other groups. In contrast, *Staphylococcus* showed a relatively low abundance in the intestinal microbiota of CcAp, CcHa, CcHc, and CcSe but a higher abundance in CcSf. Furthermore, the gut microbiota of CcAp demonstrated higher expression levels for each genus of microorganisms compared with the other four samples. Meanwhile, the gut microbiota of CcSf displayed lower expression levels for most genera, with a few exceptions (Figure 5A).

The heatmap in Figure 5B illustrates the relative abundances of the top 20 OTUs among the five samples. The expression levels of these 20 operational taxonomic units (OTUs) in CcAp were relatively high, while the abundance of OTUs in CcSf was comparatively lower than that observed in other samples, except for specific OTUs. The abundances of OTU813 (*s_Staphylococcus_saprophyticus_g_Staphylococcus*) and OTU1111 (*s_Staphylococcus_sciuri*) in CcSf exhibited a significantly higher level than the other samples.

### 3.5. Correlation Analysis of Intestinal Microbial Abundance and Development of C. cunea

**Figure 6:**
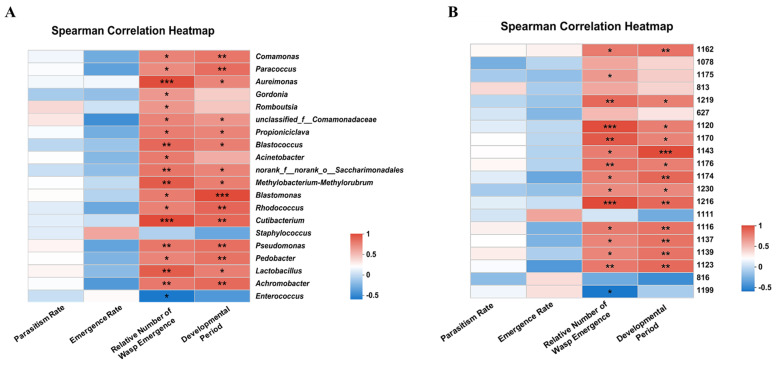
The Spearman correlation heatmap shows the relationship between intestinal microbial abundance and development among CcHc, CcAp, CcHa, CcSe, and CcSf for the 20 most abundant genera (**A**) and OTUs (**B**). The *X*-axis represents development-related factors, while the Y−axis represents species/OTUs. The correlation coefficient (R−value) and significance level (*p*−value) were calculated to assess their relationship. * 0.01 < *p* ≤ 0.05, ** 0.001 < *p* ≤ 0.01, and *** *p* ≤ 0.001. Different colors in the figure indicate varying R values. The color legend on the right illustrates intervals for different R values.

At the genus level, *Cutibacterium* and *Aureimonas* exhibited a robust positive correlation with the relative number of wasp emergence while showing no discernible association with the overall abundance of these genera in each sample. The relative number of wasp emergence exhibited a moderately positive correlation with *Pseudomonas*. A significant corresponding relationship was observed between the abundance of this genus in each sample. The correlation between *Blastomonas* and the developmental period was strongly positive, while no significant correlation was observed between *Blastomonas* and the overall abundance of this genus in all samples. *Comamonas* had a moderate positive correlation with the developmental period and a negative correlation with the abundance of this genus in each sample (Figure 5A and Figure 6A).

As depicted in Figure 5B and Figure 6B, at the OTU level, OTU1216 (*s_Cutibacterium_acnes_g_Cutibacterium*) and OTU1120 (*s_Lactobacillus_brevis_g_Lactobacillus*) exhibited a robust positive correlation with the relative number of wasp emergences; however, they did not show any significant correlation with the abundance of this genus in each sample. OTU1162 (*s_uncultured_ bacterium_g_Aureimonas*) displayed a moderately positive correlation with the relative number of wasp emergence and a significant correlation with the abundance of this genus in each sample. Meanwhile, OTU1143 (*s_uncultured_bacterium_g_Blastomonas*) demonstrated a strong positive correlation with the developmental period but showed no apparent association with the abundance of this genus across all samples. Lastly, OTU1139 (*s_Lactobacillus_fermentum*) had a moderately positive correlation with the developmental period but exhibited an evident negative correlation with its own abundance across all samples.

## 4. Discussion

Parasitic insects typically select their host based on various signals and factors to ensure successful parasitization and development of their offspring. For instance, they may utilize olfactory perception of host odor information compounds for host selection. Our preliminary research demonstrated that *Hy. cunea* pupae exhibit significantly higher attraction to *C. cunea* than *A. pernyi* pupae and *He. armigera* pupae. Meanwhile, *He. armigera* pupae show significantly higher attraction to *C. cunea* than *S. exigua* pupae and *S. frugiperda* pupae [17,21]. In this study, we observed that although the parasitization rate and emergence rate of *C. cunea* were not significantly different after parasitizing five different host pupae, there was a significant difference in the number of emerged adults and the duration of developmental stages among them (Figure 1). CcAp had the highest number of emerged wasps, followed by CcHc. The duration of developmental stages also showed significant differences, with CcSf being the shortest, followed by CcHc and CcHa (Figure 1B). Based on these findings regarding the emerged adult count and developmental stage duration, we speculate that *Hy. cunea* and *A. pernyi* are optimal hosts for *C. cunea* regarding reproduction output [17].

Intestinal microorganisms and hosts have a mutually beneficial symbiotic relationship [22]. Microorganisms significantly influence the behavior, reproductive output, and development of the host, while the structure and diversity of intestinal microorganisms vary depending on environmental conditions and food sources within the host’s habitat [23,24]. For instance, the gut microbiota of larvae from different pear orchards belonging to the same moth species exhibit variations; however, the gut microbiota did not differ between two moths collected from the same host plant [8]. The food preference of insects affects their life cycles, while the characteristics of plants as host food sources impact pest quantities [24]. Feeding oriental fruit moths with various host plants (apples, peaches, nectarines, Asian pears, plums, and apricots) revealed that *G. molesta* feeding on plums displayed the highest limited growth rate (λ), shortest doubling time (td), and greater richness and diversity in gut microbiota composition [9]. Our experiments also discovered that the wasps emerging from *Hy. cunea* and *A. pernyi* pupae had a relatively higher number of emerging wasps than other samples while simultaneously exhibiting richer gut microbiota compositions (Figure 1 and Figure 4).

Our experiments found significant differences in the relative emerged adult counts for *C. cunea* reared by different host pupae, and distinct structures were observed in their respective intestinal microbiotas. Firmicutes was identified as an advantageous phylum for CcSf, whereas it was both Firmicutes and Proteobacteria for CcHa, CcHc, and CcSe, respectively. Proteobacteria was dominant for CcAp, while Firmicutes ranked second among phyla for this group (Figure 4A). This outcome aligns with findings from studies conducted on other insects where Firmicutes and Proteobacteria were identified as the predominant phyla of the intestinal microbiota in parasitic wasps *N. vitripennis* [14]. Many studies have demonstrated that the composition of insect intestinal microbiota can be influenced by the host species and dietary habits [10,25,26,27,28]. However, these microbes may be environmentally acquired and transient, not limited by the host. Our findings are consistent with those of other insects. For instance, Firmicutes is the predominant phylum in the intestines of *Hy. cunea*, *S. exigua*, and *S. frugiperda*, while Proteobacteria ranks as the second largest phylum [24,29,30]. Firmicutes replaces Proteobacteria as the dominant phylum following nucleopolyhedrovirus (HearNPV) infection in *He. Armigera* [31]. Similarly, Proteobacteria supersedes Firmicutes as the dominant phylum under high- and low- temperature stress in *S. exigua* [29].

Comparatively, CcAp exhibited a more diverse composition of intestinal microbiota, with *Achromobacter* being its predominant genus, at only 31.8%. Conversely, CcSf displayed a highly uniform composition, with *Enterococcus* accounting for an impressive 88.7% dominance (Figure 4B). The diversity of intestinal microbiota composition in CcHc came second to that of CcAp. *Enterococcus* was the dominant genus in CcSf, CcHa, CcHc, and CcSe (Figure 4B). Following previous reports, *Enterococcus* is abundant in the intestines of Lepidoptera insects and is also a prominent genus within *S. frugiperda* and *Hy. cunea* [24,28,30,32]. The intestinal symbiotic bacterium HcM7, a member of the *Enterococcus* genus, enhances the expression of the HcGlv1 gene, inhibits the replication of HycuNPV (*Hy. cunea* nucleopolyhedrovirus), and, consequently, enhances the post-infection survival rate of *Hy. cunea* larvae [22]. However, *Enterococcus* plays a crucial role in insecticide resistance among diamondback moths [33]. At the species level, *Enterococcus mundtii* is the predominant species in CcSf, accounting for 53.3%, followed by 31.9% in CcHa samples (Figure S5). This finding has prompted our focus on researching the progress of *Enterococcus mundtii*. However, reports indicate that *Enterococcus mundtii* E14, isolated from the gut of chlorantraniliprole (CAP)- resistant *Tuta absoluta*, exhibits high CAP tolerance and can degrade CAP into non-toxic compounds, thereby enhancing *T. absoluta*’s resistance to CAP [34]. The presence of *Enterococcus mundtii* demonstrates antagonistic effects on *Hy. cunea* mortality caused by nuclear polyhedrosis virus (NPV) and *Bacillus thuringiensis* Berliner (Bacillales: Bacillaceae) (Bt) [35]. Moreover, *Enterococcus mundtii* significantly promotes early-instar *Conogethes punctiferalis* larvae growth by enhancing body length, weight, digestive enzyme activity, and protein levels, thus highlighting its crucial role in insect development [36]. *Achromobacter* was the most abundant genus in CcAp samples, but no specific category could be identified at the species level (Figure S4). Among them, *unclassified_g__Achromobacter* accounted for 31.8% in CcAp, followed by CcSe (20.8%), CcHa (15.3%), and CcHc (10.5%). *Achromobacter xylosoxidans* can degrade and utilize a variety of pesticides (such as malathion, thiacloprid, and chlorfenapyr) as a nutritional source for its growth, thereby enhancing the resistance of *Tribolium castaneum* to these pesticides [36].The *Achromobacter spanius*, isolated and cultured from wild *Drosophila suzukii*, exhibits a dose-dependent lethal effect on both *Drosophila suzukii* and *Drosophila melanogaster* [37].Whether any of these bacterial genera impact the developmental period of *C. cunea* requires further investigation. For instance, the gut microbiome can confer protection to the host against pathogen invasion through a range of mechanisms collectively referred to as ‘colonization resistance’, which may constitute a contributing factor [38]. Therefore, further investigations such as in vitro culture and identification of these intestinal bacteria, as well as the use of antibiotics to eliminate them to analyze their potential impact on the development of *C. cunea*, need to be conducted.

The analysis of intestinal microbial abundance and development of *C. cunea* revealed a moderate positive correlation between *Pseudomonas* and OTU1162 (*s_uncultured_bacterium_g_Aureimonas*) with the relative number of wasp emergence. Similarly, *Trichogramma chilonis*, a parasitic natural enemy insect, exhibited a significant increase in the mean female ratio and enhanced the parasitization of *He. armigera* eggs when provided with a diet containing *Pseudomonas* sp., although *Pseudomonas* species are emerging as significant human pathogens worldwide [39,40]. A clear corresponding relationship was observed between the abundance of this genus in each sample. *Comamonas* and OTU1139 (*s_Lactobacillus_fermentum*) exhibited a moderately positive correlation with the developmental period while showing a significantly negative correlation with the abundance of bacteria belonging to this genus in each sample (Figure 6). However, further investigation is required to determine whether these bacterial genera and OTUs are associated with the relative number of wasp emergences or the developmental period, such as via colony isolation and culture.

## 5. Conclusions

In summary, our findings suggest that the intestinal microbiota of *C. cunea* exhibits a higher number of OTUs after parasitizing *Hy. cunea* pupae while demonstrating a greater species count following parasitization in *A. pernyi* pupae. The community of intestinal microbes in *C. cunea* displays increased diversity and more extensive metabolic pathways when parasitizing *A. pernyi* pupae. The diversity of the intestinal microbiota and enzymatic activity within it may contribute to wasp development and enhance adult emergence counts. However, further investigation is required to elucidate the role of the intestinal microbiota in the wasps’ development and immune system, such as isolating key bacteria from the intestinal canal of *C. cunea* to identify those promoting wasp emergence and detecting metabolite differences during parasitization in different host pupae species.

## Data Availability

The data presented in this study are openly available in [FigShare] at https://figshare.com/articles/dataset/dx_doi_org_10_6084_m9_figshare_6025748/6025748.

## Supplementary Materials

The following supporting information can be downloaded at: https://www.mdpi.com/article/10.3390/insects15090722/s1, Figure S1: The rarefaction curves of gut microbiota in *C. cunea* among different hosts. Figure S2: The co-occurrence relationships of core bacteria among CcHc, CcAp, CcHa, CcSe and CcSf. Figure S3: The number of categories among CcHc, CcAp, CcHa, CcSe and CcSf. Figure S4: In the Venn diagram, different colors represent different samples; the numbers of overlaps and folds represent the shared and the unique species numbers, respectively. Figure S5: Column plots show the bacterial community composition at the species level across different samples. Table S1: The sequencing data statistics for all samples. Table S2: Summary table of OTU abundance and classification information. Table S3: The comprehensive data for the Venn diagram at the genus level.
