# Supplementary material for: Host Species Affects Gut Microbial Community and Offspring Developmental Performances in the Pupal Parasitoid Chouioia cunea Yang (Hymenoptera: Eulophidae)
Source: Insects. 2024 Sep 20;15(9):722. doi: 10.3390/insects15090722 (PMC11432438; doi:10.3390/insects15090722)
Supplement: Supplementary file 1 [file insects-15-00722-s001.zip › Table S and Figure S/Figure S.pptx]

## Slide 1
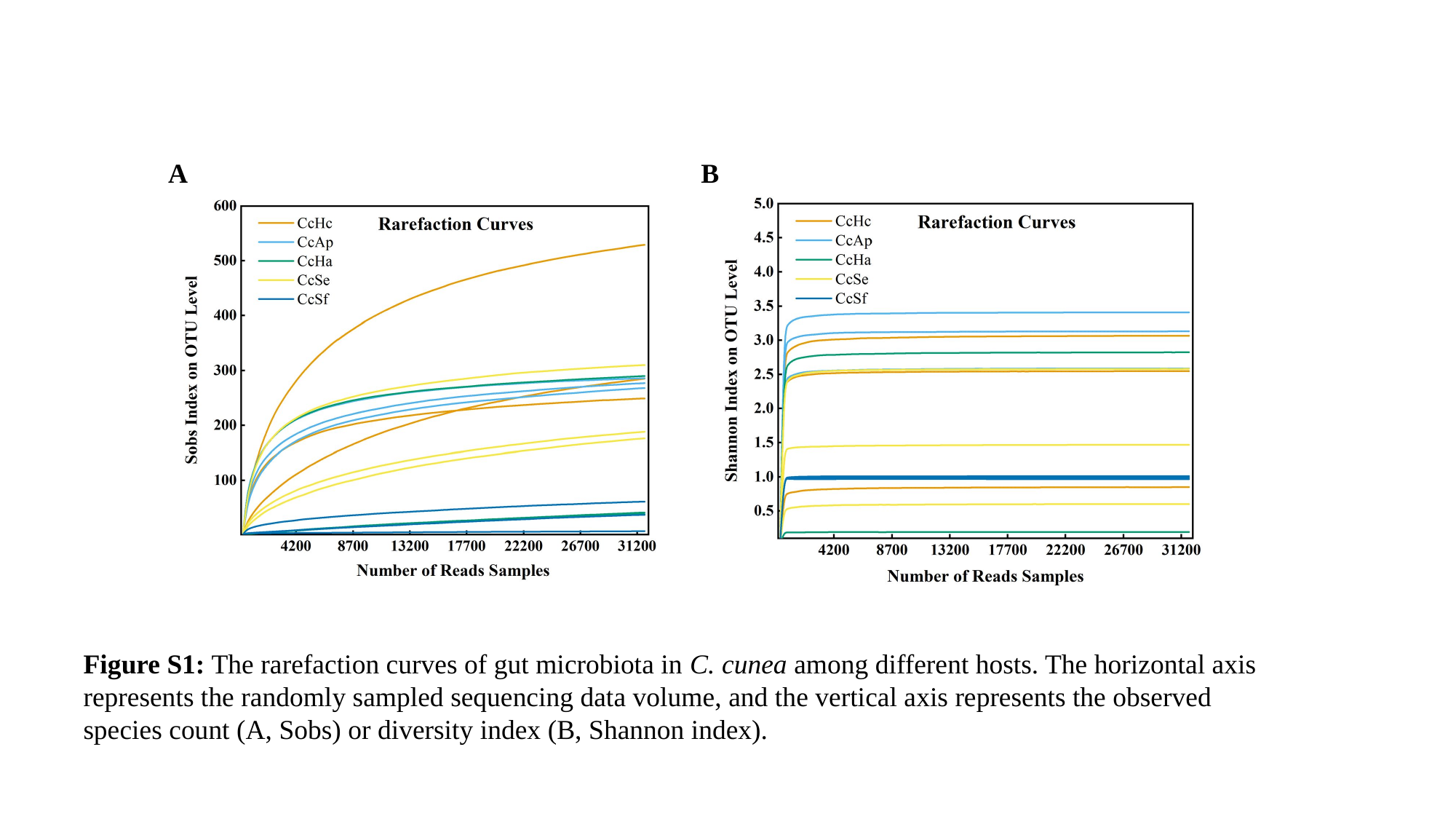

A
B
Figure S1: The rarefaction curves of gut microbiota in C. cunea among different hosts. The horizontal axis represents the randomly sampled sequencing data volume, and the vertical axis represents the observed species count (A, Sobs) or diversity index (B, Shannon index).

## Slide 2
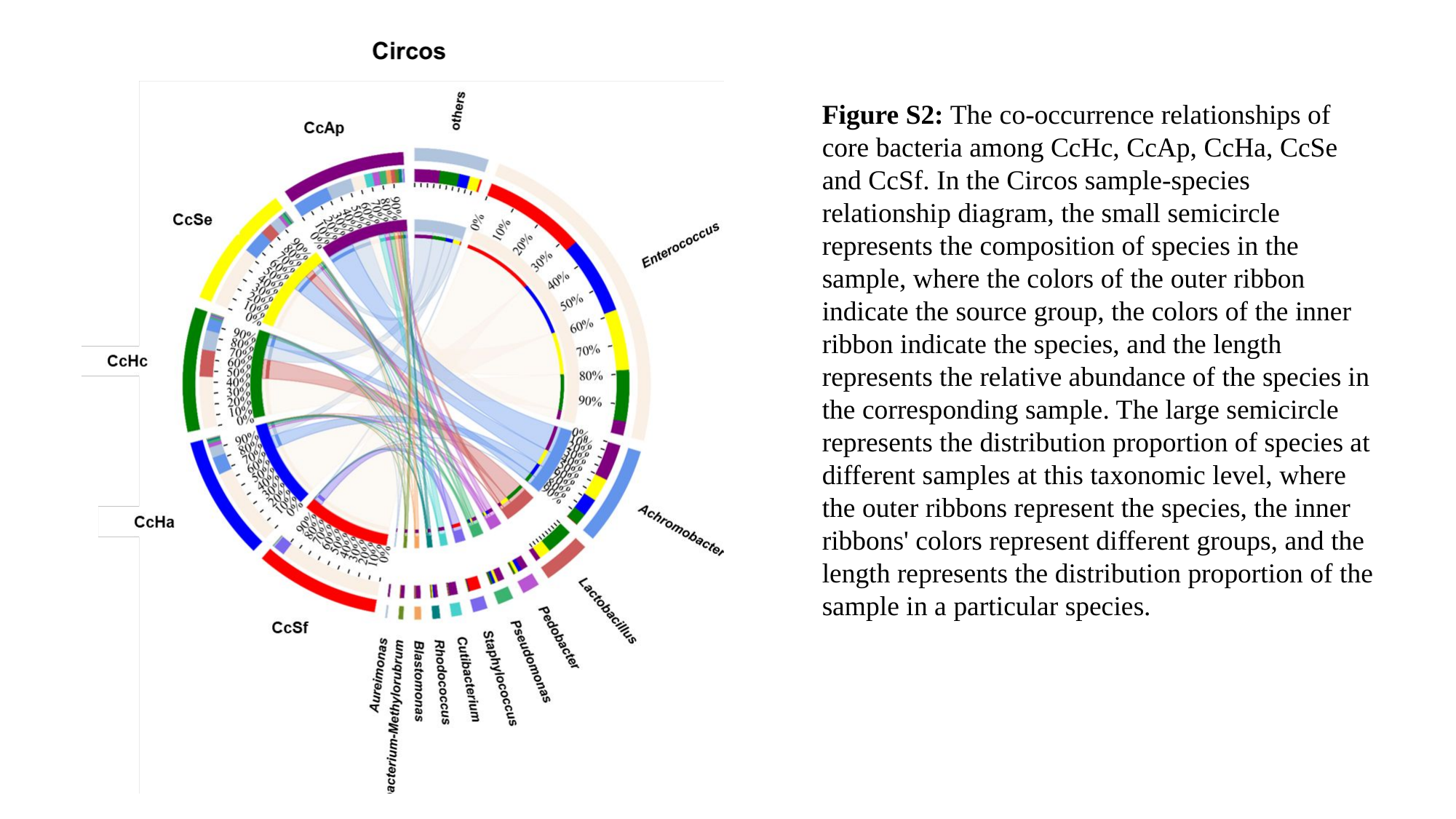

Figure S2: The co-occurrence relationships of core bacteria among CcHc, CcAp, CcHa, CcSe and CcSf. In the Circos sample-species relationship diagram, the small semicircle represents the composition of species in the sample, where the colors of the outer ribbon indicate the source group, the colors of the inner ribbon indicate the species, and the length represents the relative abundance of the species in the corresponding sample. The large semicircle represents the distribution proportion of species at different samples at this taxonomic level, where the outer ribbons represent the species, the inner ribbons' colors represent different groups, and the length represents the distribution proportion of the sample in a particular species.

## Slide 3
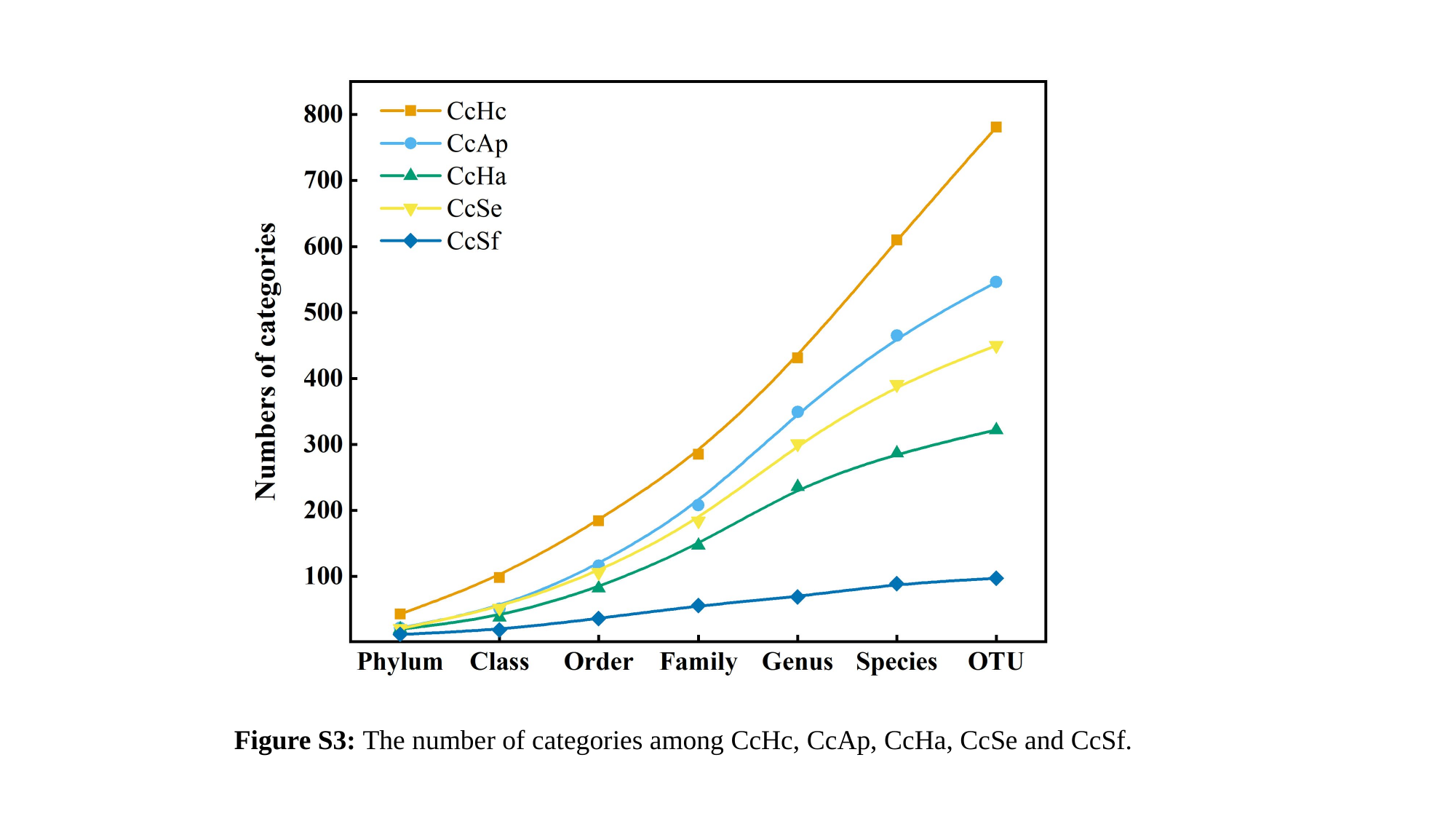

Figure S3: The number of categories among CcHc, CcAp, CcHa, CcSe and CcSf.

## Slide 4
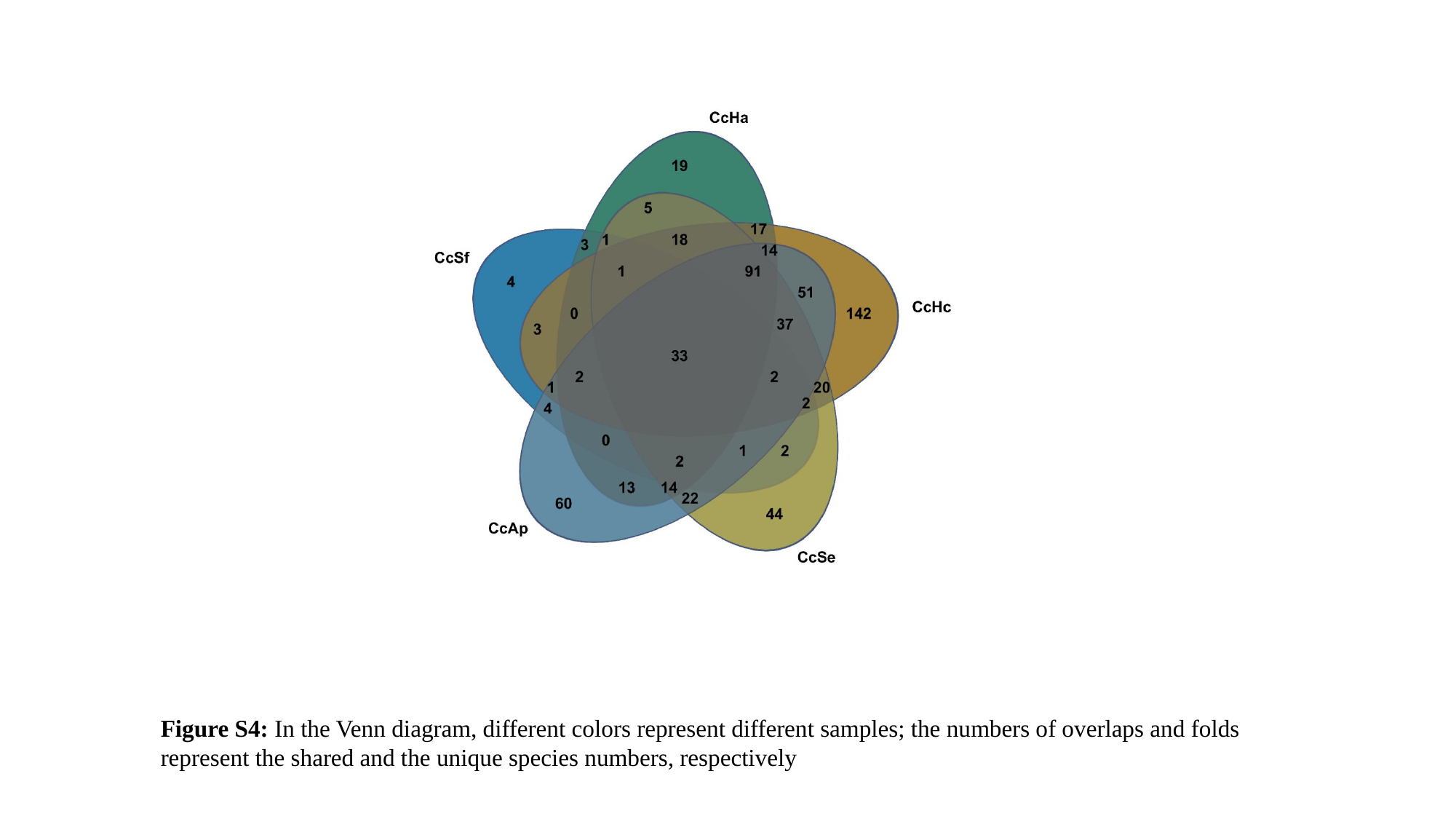

Figure S4: In the Venn diagram, different colors represent different samples; the numbers of overlaps and folds represent the shared and the unique species numbers, respectively

## Slide 5
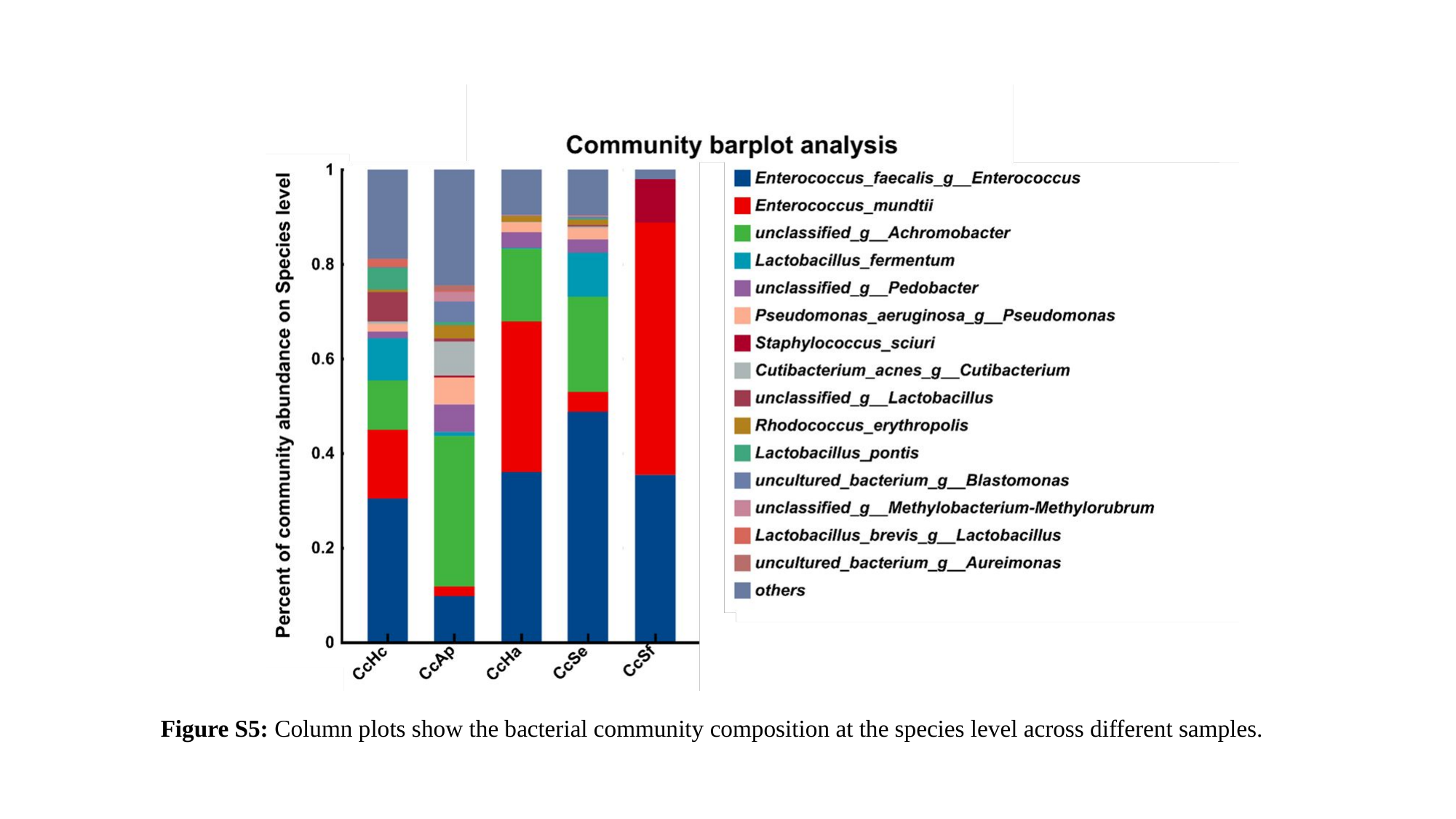

Figure S5: Column plots show the bacterial community composition at the species level across different samples.
